# P28 - Wells’ syndrome (or Eosinophilic Cellulitis) – a case report

**DOI:** 10.1186/2045-7022-4-S1-P83

**Published:** 2014-02-28

**Authors:** Dimitrios Koutsalitis, Dimitrios Karantoumanis, Anastasios Konstantinopoulos, Anna Pananaki, Maria Psomiadou, Kassiani Tzeli, Marianna Tziotou, Eirini Roumpedaki, Nikolaos Douladiris, Emmanouil Manousakis, Nikolaos G Papadopoulos

**Affiliations:** 1 Allergy Department, 2nd Pediatric Clinic, University of Athens, Athens, Greece

## Background

Wells syndrome belongs in diseases with eosinophilic involvement in specific organs (eg, skin, lungs).

Peripheral eosinophilia (mild or moderate-profound) is present in more than 50%. May be idiopathic, associated with drugs, or even associated with myeloproliferative, immunological or infectious diseases. Reported about 80 incidents worldwide.

Has an excellent prognosis. It tends to resolve in weeks or months, usually without scarring. It occasionally recurs. In these recurrent cases, it can take years to ultimately resolve.

## Case report

We present a 7 ½ year’s old girl with a history of peripheral eosinophilia (range of Eo: 766-2580) and skin cellulitis. Mild itching affected area. Not general symptomatology.

Personal and family history: free.

During the hospitalization was carried out frequent laboratory and clinical control.

From the above laboratory and diagnostic work out, finding of dubious Abs for Toxocara and CRP (ranging from 3-12), is not found something important.

Myelogram also was carried out without evidence.

Throughout hospitalization the child was under antibiotic treatment.

Each skin inflammation will subside after the lapse of about 10 days followed by hyperpigmentation lasting about one month.

The diagnosis will come eventually with skin biopsy.

By far regular monitoring of the child (1½ year from the close of hospitalization) are not observed any organ damage, although the absolute number of Eo will fluctuate between 260 and 1340 cells.

During the first 4 months after the last treatment the child will be implemented by our department 3 times short courses (lasting 4-7 days) treatment p.os K/S (dose 1-1,5mg/kg) in exacerbations syndrome (peripheral eosinophilia and skin cellulitis) with spectacular resolved within 4 to 5 days so as eosinophilia and skin lesions. Since then in complete remission.

## Discussion

We consider the case as idiopathic. So far with excellent prognosis.

## Noteworthy

The “preference” for recurrence of cellulitis at sites of initial localization and no in new positions.

